# Asymmetric parameter enhancement in the split-ring cavity array for virus-like particle sensing

**DOI:** 10.1364/BOE.483831

**Published:** 2023-02-22

**Authors:** Xiao Jin, Lu Xue, Shengwei Ye, Weiqing Cheng, Jamie Jiangmin Hou, Lianping Hou, John H. Marsh, Ming Sun, Xuefeng Liu, Jichuan Xiong, Bin Ni

**Affiliations:** 1 School of Electronic and Optical Engineering, Nanjing University of Science and Technology, Nanjing 210094, China; 2 James Watt School of Engineering, University of Glasgow, Glasgow, G12 8QQ, UK; 3 Department of Medicine, University of Cambridge, Hills Road, Cambridge, CB2 0QQ, UK; 4 Co-first authors; 5 Co-last authors; 6 jichuan.xiong@njust.edu.cn

## Abstract

Quantitative detection of virus-like particles under a low concentration is of vital importance for early infection diagnosis and water pollution analysis. In this paper, a novel virus detection method is proposed using indirect polarization parametric imaging method combined with a plasmonic split-ring nanocavity array coated with an Au film and a quantitative algorithm is implemented based on the extended Laplace operator. The attachment of viruses to the split-ring cavity breaks the structural symmetry, and such asymmetry can be enhanced by depositing a thin gold film on the sample, which allows an asymmetrical plasmon mode with a large shift of resonance peak generated under transverse polarization. Correspondingly, the far-field scattering state distribution encoded by the attached virus exhibits a specific asymmetric pattern that is highly correlated to the structural feature of the virus. By utilizing the parametric image sinδ to collect information on the spatial photon state distribution and far-field asymmetry with a sub-wavelength resolution, the appearance of viruses can be detected. To further reduce the background noise and enhance the asymmetric signals, an extended Laplace operator method which divides the detection area into topological units and then calculates the asymmetric parameter is applied, enabling easier determination of virus appearance. Experimental results show that the developed method can provide a detection limit as low as 56 vp/150µL on a large scale, which has great potential in early virus screening and other applications.

## 1. Introduction

In recent years, bioparticles such as viruses and nanoplastics have raised great concerns due to their low refractive indexes and are only tens of nanometers in size [1–3]. Increasing water pollution and the global COVID-19 pandemic have demanded higher requirements for simple, rapid, accurate and highly sensitive detection methods for virus-like particles.

For the detection of virus-like particles with a relatively low concentration, various methods have been presented. Typical labeling methods for rapid virus detection include enzyme-linked immunosorbent assay (ELISA) [4,5] and colloidal gold [6,7]. Other methods include Fourier-transform infrared spectroscopy (FTIR) [8,9], ultraviolet-visible spectroscopy (UV-ViS) [10,11] and Raman scattering (RS) [12,13], which utilize the scattering spectrum as characters to determine the existence of viruses. Nevertheless, signal enhancement methods are necessary for virus-like particle samples with low concentrations.

Polymerase Chain Reaction (PCR) utilizes fluorescent dyes and is the most commonly used detection method for viruses with low concentration [14–17]. PCR can characterize coronaviruses with a concentration of about 100-1000 copies/mL [18,19]. However, complex sample treatment processes and expensive laboratory equipment limit its wide application.

To enhance the optical signals of virus-like particles, the plasmonic effect could also be utilized to detect virus-like particles with low concentrations [20,21]. Surface-enhanced fluorescence (SEF) takes advantages of the amplification of fluorescence at the localized hot spot. Fluorescent labels are still necessary for marking target virus-like particles [22–24]. Similar to SEF, surface-enhanced Raman scattering (SERS) strengthens the signals of Raman scattering and analyzes the response on scattering spectrums to detect specific virus-like particles [25,26]. During COVID-19, numerous detection methods for coronavirus based on SERS are proposed [27–31]. However, quantification is always a problem for the application of SERS to a more detailed analysis of virus-like particles [32]. A rapid quantifiable detection method is in exceptional demand.

Recently, we proposed a virus-like particle detection method based on gold nanodots array on the Si substrate [33]. The spatial symmetry of the structure and dielectric environment of the nanodots will be broken when a virus-like particle appears in its vicinity, and the corresponding polarization parameters of the scattered light in the far field would therefore generate asymmetric patterns, which are utilized for quantitative analysis. Theoretically, a detection radius of 150 nm around the gold nanodot could be achieved based on our protocol. However, in the experiment, a complex optical environment due to the imperfection of nanodots and impurities in the sample would highly reduce the detection radius. Due to the small area of contact spot between the particle and the nanodot, particles could hardly attach to gold nanodots. Also, as a result of the rotational symmetry of the nanodot with respect to an axis normal to the substrate surface, virus-like particles could attach to the nanodot from random directions with respect to the rotational axis of the gold nanodot, leading to that the measured polarization parametric images have random distributions and are complicated to analyze.

**Fig. 1.**
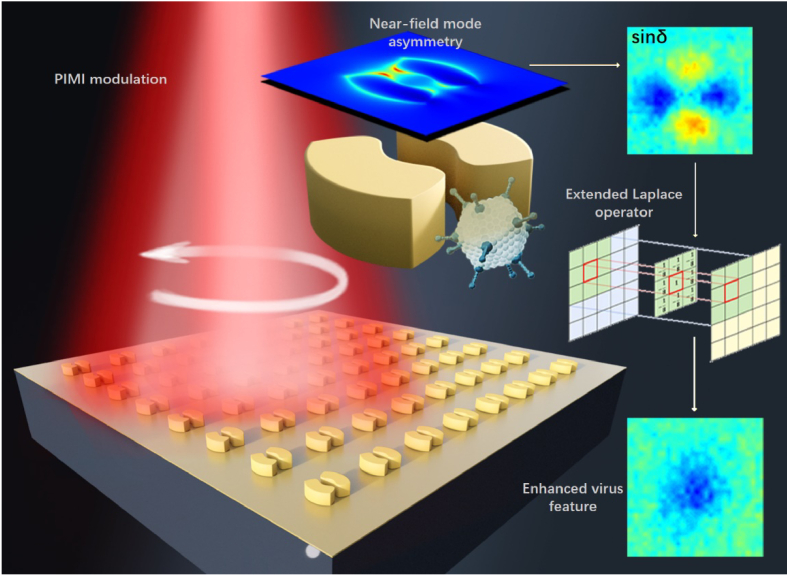
Diagram of detection for virus-like particle based on the split-ring structure.

In this article, a new plasmonic array was designed and fabricated, with a split-ring structure as the basic element which has a symmetric plane along the splitting gap and is normal to the substrate surface. As shown in Fig. 1, The large area of contact at the two splitting gaps provides a higher possibility for viral-like particles to attach to the nanodots at a specific orientation. From the near-field simulations based on the Finite Difference Time Domain (FDTD) method, a strong coupling of electric field on the one side of the split-ring occurs when an adenovirus is attached to the gap.

To be noticed, a metal layer with a thickness of 10 nm is then deposited on the top surface of split-ring structures and viral-like particles to enhance the plasmonic asymmetry signals caused by the attachment event. Au is used as the enhancement layer in this case for convenience. Considering the cost and reuse of the plasmonic array, we could use some other material as the enhancement layer, which could also be removed easily.

The experiment is implemented in a Polarization Indirect Microscopic Imaging (PIMI) system, which could obtain high-resolution parametric images that is highly sensitive to subwavelength nanostructures [34,35]. Based on the periodic arrangement of nanodot elements, an extended Laplace operator is proposed for image processing. After the convolution between the operator and polarization parametric images, the output results show a high sensitivity to the split ring conjugated with viruses. A subsequent calculation of asymmetry is performed to quantify the image variation caused by the virus and the possibility and confidence of virus presence at a certain detection unit, i.e., a single nanodot, could be quantified. When a regular sample volume is applied, the detection limit could reach up to 56 vp/150µL (vp = virus particle).

## 2. Experimental and simulation setup

### 2.1 Sample preparation

**Fig. 2.**
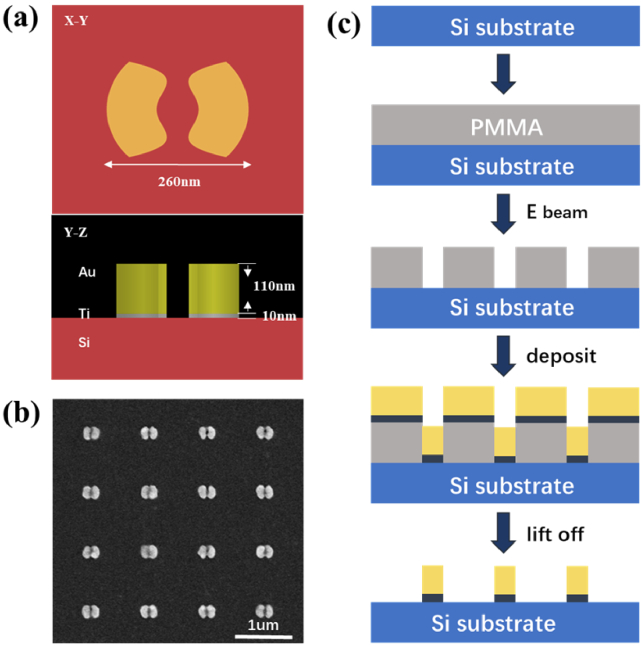
(a) Cross sections of the split-ring structure, (b) SEM image of fabricated split-ring structures and (c) the flow chart of fabrication processes.

The plasmonic structure of the split-ring nanodot array and the fabrication process is shown in Fig. 2(a) and 2(c), respectively. A subwavelength Ti/Au split-ring nanodot array is fabricated for anchoring virus-like particles. Si substrate was cleaned by acetone and isopropyl alcohol (IPA) solvents first with a water bath at a temperature of 50°C. After ultrasonic cleaning, a 200 nm thick layer of Polymethyl Methacrylate (PMMA) photoresist was spun onto the surface of the silicon sample by controlling the spinning speed.

Electron beam lithography (EBL) was used to define the patterns on the PMMA resist with a high resolution. After development, the gold nanodot array pattern was transferred to PMMA. Next, a 10 nm thick titanium layer and a 110 nm thick gold layer were deposited successively on the surface of the silicon sample by the Electron Beam Evaporation method. The 10 nm thick titanium was first deposited mainly to enhance the metal adhesion with the silicon surface. After a standard lift-off process, the gold nanodot array was finally realized.

Considering fabrication difficulty, two smooth curves with a ω-like shape were used to split the gold disk as the resonant cavity. The function of curves is $\text{y}=0.0000015*{\left({\text{x}}^{2}-3600\right)}^{2}+20$ , which possesses a central hole slightly smaller than the virus size. The interval between two adjacent split-ring nanodots in the horizontal and vertical directions is 1 µm to avoid excessive plasmonic crosstalk and image overlap. The fabricated structures are clarified by a scanning electron microscope (SEM) as shown in Fig. 2(b).

In the experiment part of this work, adenovirus was used as the virus-like particle sample for detection. Adenovirus is a type of icosahedral virus with a uniform size of around 80 nm, which is a suitable size for our designed structure. The virus (obtained from Hanbio Technology Co., Ltd) was dissolved in phosphate buffer saline (PBS) and a microdroplet of about 1 µL was dropped directly onto the split-ring array, and then it was ready for detection after drying.

### 2.2 PIMI system

The main optical path of the system is built based on an Olympus BX51 microscope. A liquid crystal tunable filter (Thorlabs KURIOS-WB1) associated with a quarter wave plate is placed in front of a halogen lamp source to select the illumination wavelength with a bandwidth of 35 nm. In the PIMI system, the light source is continuously modulated with a polarization angle step of 18° by a rotating polarizer. A quarter-wave plate and a linear analyzer with angles of 45° and 90° with respect to the X-axis are placed respectively at the reflection light path. A highly sensitive charge-couples sensor (CCD, PiA2400-17gm, Basler) with 5 million pixels combined with a 100× objective lens (NA = 0.9) is used to record the light intensity images under different polarization states (diagram of PIMI system is shown in Fig. S1 in the Supplement 1).

In PIMI measurement, each pixel intensity variation of the complete modulation process can be expressed as:

(1) $$I_{i}=\frac{1}{2}I_{0}[1+sin\delta sin2(\theta_{i}-\phi )],$$

where $I_{i}$ (the subscript *i* represents the number of polarization rotation angles) indicates the pixel intensity. $I_{0}$ is the average intensity under all polarization states. sinδ represents the sine of the phase difference between two orthogonal polarization components. $\theta_{i}$ is the polarization angle of the linearly polarized incident beam, and ϕ is the polarization ellipse orientation angle of the reflected beam from the sample (more details of the theory and calculation of PIMI are in the Supplement 1).

### 2.3 Image processing

**Fig. 3.**
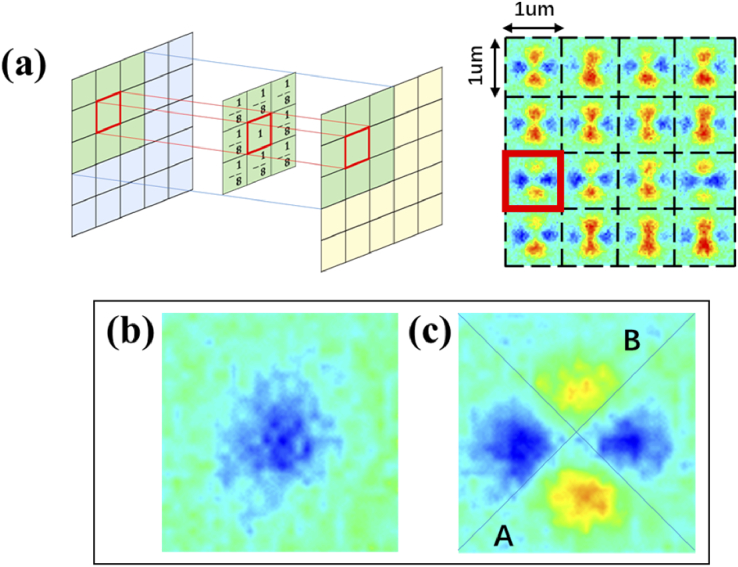
Data processing template division method. (a) diagram of extended Laplace operator applied on raw sinδ image units, (b) convolution results of extended Laplace operator on part of the sinδ image unit, (c) segmentation of a sinδ unit for calculation of asymmetry

To characterize the asymmetric feature of the split-ring with the attached virus, an extended Laplace operator is employed as the convolutional kernel for image processing. As shown in Fig. 3(a), the obtained image is segmented into topological units with a size of 1 µm × 1 µm, centered on the centers of split-ring structures.

Every nine adjacent split-rings are then combined as an extended Laplace operator. Instead of a larger period, the minimum period of the split-ring is defined as the pixels in the traditional Laplace operator to avoid being influenced by potential background unevenness far away from the center topological unit. The unit value is shown below:

(2) $$\begin{array}{c} \text{I}(\text{i},\text{j})={\text{I}}_{0}(\text{i},\text{j})-\frac{1}{8}(({\text{I}}_{0}(\text{i}-1,\text{j}-1)+{\text{I}}_{0}(\text{i},\text{j}-1)+{\text{I}}_{0}(\text{i}+1,\text{j}-1)+{\text{I}}_{0}(\text{i}-1,\text{j})+\\ {\text{I}}_{0}(\text{i}+1,\text{j})+{\text{I}}_{0}(\text{i}-1,\text{j}+1)+{\text{I}}_{0}(\text{i},\text{j}+1)+{\text{I}}_{0}(\text{i}+1,\text{j}+1)). \end{array}$$

Here i and j represent unit numbers along the X-axis and Y-axis respectively. ${\text{I}}_{0}$ contains raw values in sinδ images, and I is the output image after convolution. This parameter could reveal an intensity decrease for PIMI image caused by a virus coated with Au. Also, as shown in Fig. 3, to quantitively clarify the asymmetry caused by virus binding for each unit, we define asymmetry values as a combined parameter for changes in intensity and symmetry. As shown below:

(3) $$As_{sin\delta }={\text{I}}_{\text{sum}}\cdot (A/B+B/A\;-2),$$

where A and B are the sums of intensities in the two parts in Fig. 3(c), and ${\text{I}}_{\text{sum}}$ is the summed values in Fig. 3(b) with intensities higher than 0 in one unit.

### 2.4 Simulation based on FDTD

Near-field electric field features are simulated based on the finite difference time domain (FDTD) method to investigate the coupling between the split-ring and the virus coated with a thin Au layer. This simulation is conducted via the commercial software Lumerical FDTD.

The lengths of the simulation region along the X-axis, Y-axis, and Z-axis are set as 4 µm, 4 µm, and 2 µm respectively. All the boundaries are set as Perfectly Matched Layer (PML). 10-nm-Ti and 110- nm-Au split-ring structures with an outer radius of 130 nm are first put on a silicon substrate. To simulate the experimental environment, a virus with a diameter of 80 nm and refractive index of 1.47 is attached to the top gap of the split-ring. In addition, we set a layer of protein (refractive index of 1.47) at the center of the split-ring with a thickness of 60 nm and a longitudinal length of 140 nm. An Au layer with a thickness of 10 nm is then deposited onto the substrate to cover the virus and enhance its localized field. We use an Au half ellipsoid with the same radius as the virus at the X-Y surface, and a radius of 60 nm at the Z coordinate to cover the virus, considering a localized accumulation of gold may result in a thicker deposition.

Total-Field Scattered-Field (TFST) source covers the whole structure in our simulation to achieve scattering results. One monitor is placed at the top surface of the split-ring structure to investigate the asymmetry and mode distribution of the near-field electric field caused by a virus coated with Au. Another field monitor is placed at 220 nm height with respect to the substrate to calculate the far-field PIMI results. The results of ten polarization directions of the source are sequentially simulated with a step of 18°. Subsequent calculations are completed in Matlab under the algorithm mentioned above. We also add an intensity variation to match the change of intensity in the experiment.

## 3. Results and discussion

### 3.1 Simulation of asymmetry caused by virus coated with Au film

**Fig. 4.**
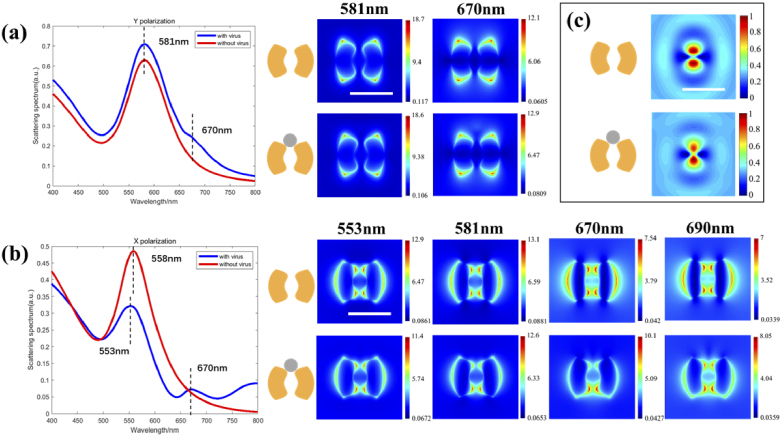
Simulated scattering spectrums and near-field electric field distributions for the split-ring attached without or with a virus under (a) Y polarized illumination and (b) X polarized illumination. (c) simulated sinδ image for the split-ring attached without or with a virus. The scale bars for (a), (b), and (c) are 250 nm, 250 nm, and 1 µm respectively.

A broadband TFSF source from the wavelength of 400 nm to 800 nm is set to characterize the scattering features of the split-ring structure. The simulated scattering spectrums for illumination under polarization at the Y-axis are shown below in Fig. 4(a). A resonant peak is shown at 581 nm for the split-ring structure. When a virus is attached, the scattering spectrum changes very little with the same peak resonant wavelength, which indicates a small influence caused by a virus. In the right part of Fig. 4(a), on the top surface of the split-ring under 581 nm, most energy is localized at four outer corners of the longitudinal direction with a symmetric shape. Due to a broadband scattering peak, this longitudinal mode could still exist at 670 nm with a weaker response. Consistent with the spectrum results, the electric field of the split-ring attached to the virus under 670 nm does not show a strong asymmetry compared with a pure split-ring.

When the split-ring is illuminated under the source with X polarization, the scattering features differ due to the resonant cavity between the two arms. In Fig. 4(b), the resonant peak for a split-ring shifts to 558 nm, where localized plasmonic enhancement in the near-field is shown at the outer surface at the transverse direction, and high energy occurs at the two split gaps. It could be induced that two gaps break the free movement of electrons, and a strong cavity resonance is generated between the two arms of the split-ring. From the right part of Fig. 4(b), the transverse mode is constant with an analogous shape within a long range of wavelength from 553 nm to 690 nm.

Considering the morphology of a virus, the main resonant peak would remain a near wavelength of 553 nm with a distinct attenuation, and the near-field mode distribution is almost the same as the bare split-ring. Nevertheless, the scattering spectrum for the transverse mode has changed significantly. An unsmoothed fluctuation is generated at a wavelength of 670 nm in the scattering spectrum, which reveals a new mode. From the electric field image, when the structure is illuminated under the peak wavelength of 670 nm, the hot spot at the side of the gap where the virus is attached vanishes, denoting that an asymmetric mode is generated by the virus coated with Au. The coated Au film provides a physical connection between two split-ring arms and breaks the condition of the symmetric cavity. The break in near-field symmetry would bring a corresponding change in the far-field sinδ image.

Based on the process in section 2.4, a simulated sinδ image is then presented in Fig. 4(c). For the bare split-ring, two strong dipoles appear symmetrically along the longitudinal direction. When the whole array is coated using gold film, an intensity asymmetry is generated along the longitudinal direction, which could match the near-field feature of the asymmetric mode in the near field. This asymmetry is shown in sinδ and is a suitable phenomenon which can be utilized for virus detection on the far field (simulation results for different sample statuses are shown in Fig. S2 and Fig. S3 in the Supplement 1).

### 3.2 Experimental results of virus detection

**Fig. 5.**
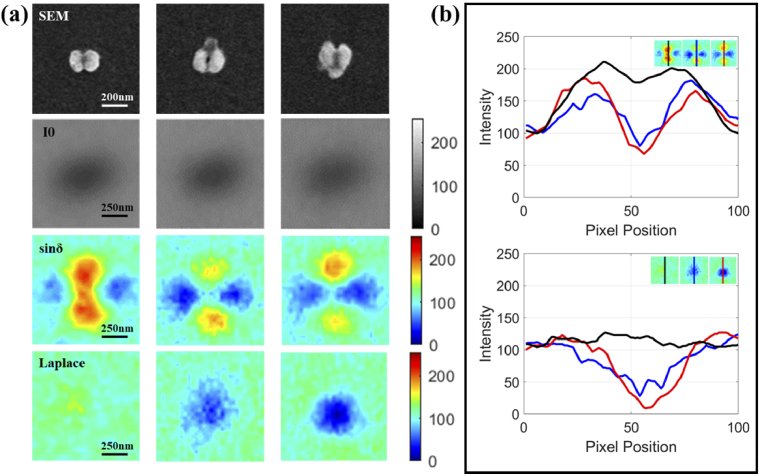
(a) Experimental results for the split-ring without or attached a virus. From top to bottom are results for SEM, images from the conventional microscope, sinδ, and extended Laplace images. (b) Intensity curves along lines axes of symmetry in longitudinal orientation for sinδ and extended Laplace images of the split-ring without or with a virus.

The experimental and processed results are shown in Fig. 5(a). Due to the diffraction limit of resolution, a lower illumination wavelength could achieve a stronger image signal. Considering the large width of the peak for the asymmetric mode caused by viruses, the experimental illumination center wavelength is set to 600 nm, where the asymmetry of the electric field is still evident in the simulation. In conventional microscope images, features of scattering fields for the split-ring and the combined structure are blurred black spots with a size of about 300 nm. Compared with similar blurred results, indirect polarized parameter sinδ images reveal more details relating to near-field mode distribution.

The sinδ image of a single split-ring shows a symmetric shape with the same feature as shown in Fig. 4(c). When the viruses are connected to one of two gaps, one side of the longitudinal dipole will be weaker than the other side, according to the effect caused by the asymmetric mode (experimental results for different sample statuses are shown in Fig. S3 in the Supplement 1). Along the midline in the longitudinal direction, three curves are displayed on the upper side of Fig. 5(b) to clarify the intensity differences. One can see that, when a virus occurs at the gap, the basic intensity of sinδ will decrease with a deeper valley in the middle. In addition, the weaker intensity peak in the curve could show the exact side the virus is present.

To reveal the pure impact caused by viruses and make a quantitative analysis, the extended Laplace operator is then used according to the process in section 2.3. At the bottom of Fig. 5(c), convolution results between sinδ topological units and the extended Laplace operator are shown at the same positions. The operator could extract and subtract a background split-ring pattern to achieve a clear result. For the bare split-ring, the dipole pattern of the split-ring in sinδ will be removed and only low intensity would remain in this area.

However, as to the extended Laplace images for the split-ring structures with viruses, strong dark spots will appear with a slight shift of center points along the longitudinal direction, which indicates the position of viruses. The same curves are drawn in the lower part of Fig. 5(b). The intensities for the split-ring without virus are corrected to a normal flat line and images for the split-ring with viruses show visible valleys with lower intensities.

**Fig. 6.**
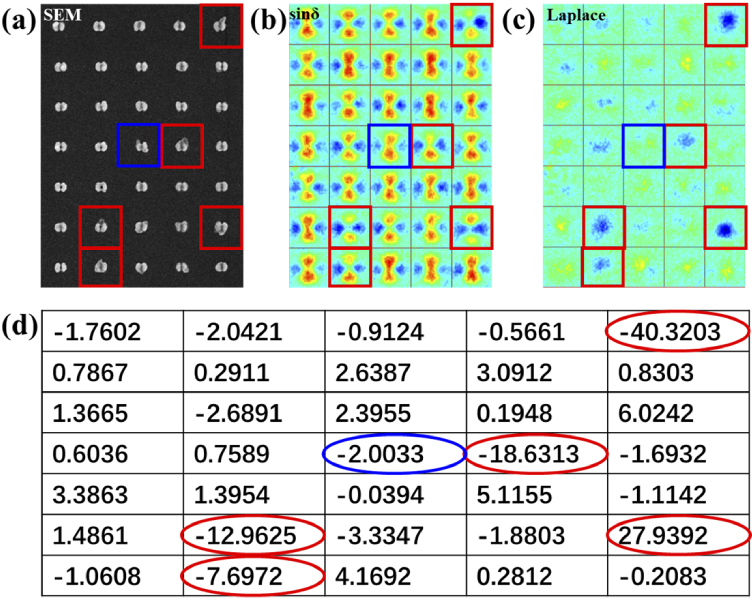
Large scale images for (a) SEM, (b) sinδ, and (c) extended Laplace. (d) Values of asymmetry corresponding to the sites in (a), (b) and (c).

To make a quantitative judgment and test the ability of our detection method, the algorithm in section 2 is applied in a typical area with several viruses attached to the split-ring structures. Based on the SEM image in Fig. 6(a), viruses are labeled in Fig. 6(b) and 6(c) with red and blue boxes. In the large scale sinδ image, the sites labeled with red boxes show good pattern characters as discussed before. When the extended Laplace operator is applied, dark spots also exist at the same site. However, due to the large fabrication errors in some structure elements or a mismatched binding position of the virus, viruses in certain locations might be missed, e.g., the virus labeled with the blue box. In the selected area, six units containing viruses are marked, and only one of them is missed due to the mismatched binding position. We could take this error into consideration when theoretically calculating the limit of detection ability.

In addition, to avoid the possibility that some sites without viruses could also generate weak signals in Fig. 6(c), a combined parameter $As_{sin\delta }$ is then calculated quantitatively to guarantee a higher detection accuracy. As shown in Eq. (3), this parameter reveals the asymmetry of each unit associated with the results of the extended Laplace using quantitative values, making the results more reliable as a judging standard. Figure 6(d) shows $As_{sin\delta }$ in a table, and the red sites containing the virus will generate large absolute values, which meets the expectation. The positive or negative sign of the values represents the direction of the virus in the near field. A threshold absolute value of 7 could be determined as the limited standard for the judgment of viruses.

### 3.3 Theoretical limit of detection ability

**Fig. 7.**
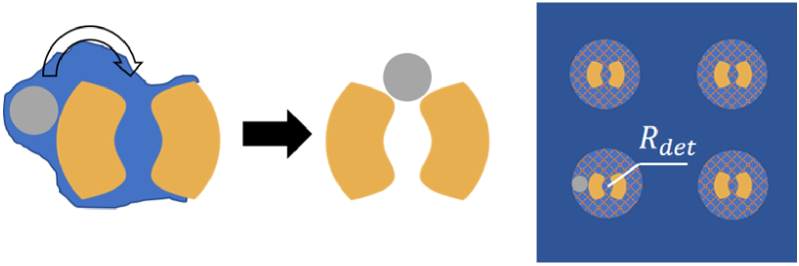
Diagram for movement of viruses when water is evaporating and the radius of detection.

As an alternative to numerous experiments for analysis of detection ability, a preliminary method based on a few experimental data could be implemented to judge the detection limit. As shown in Fig. 7, when a virus is dropped onto the substrate, there is a chance that the virus lands at a nearby area of a split-ring structure randomly. Due to the surface tension of water described by the Young–Laplace equation, the virus attached to the outer surface of split-ring structures will be pushed along the orientation of the water mask while evaporating [36]. With water evaporating, the two gaps would finally stick the virus at the positions we expected. To be noticed, the possibility of two viruses attaching to the same split-ring structure is very small, and we can ignore its influences.

Here, a parameter $R_{det}$ could clarify the radius of detection, within which the virus would be stuck into two gaps. Taking robustness into consideration, we set the scanning radius of the virus attached at the outside of the split-ring as the smallest $R_{det}=210\;nm$ . Our detection method is based on a large-scale microscopic imaging system, which allows every virus particle in the field of view to be detected. When a virus falls into the array randomly, we assume it can be detected within $R_{det}$ for a split-ring. Hence the detection possibility for single virus $P_{1}$ is

(4) $$\;\;P_{1}=\frac{\pi {R_{det}}^{2}}{L^{2}},$$

where *L* is the interval for the gold array. Considering possible misdetection caused by wrongly attached sites of several viruses or error of fabrication, a bonus possibility of misdetection $P_{2}$ is calculated from the count of undetected and detected viruses attached to split-ring structures. By comparing the SEM results and the PIMI image, $P_{2}$ could be estimated as 1/6. The attachment process of N viruses could be treated as a series of independent events. To calculate the lower limit quantity for virus detection, a parameter $P_{N}$ is defined as the possibility that at least one virus is detected by the gold array:

(5) $$P_{N}=1-C_{N}^{0}P^{0}{\left(1-P_{1}(1-P_{2})\right)}^{N}.$$

We set a threshold $P_{N}>0.999$ as the standard for a stable detecting ability, where the solved *N* is

(6) $$N>\frac{-3}{lg\left(1-\frac{\pi {R_{det}}^{2}}{L^{2}}(1-P_{2})\right)}$$

Substituting values into the formula, the minimum *N* is calculated as 56, which is much smaller than conventional results. If we apply a regular sample volume of 150 µL, the same volume as the used reference, the detection limit is 56 vp/150µL. Although a larger $R_{det}$ could theoretically decrease the limited number of viruses we need, a large structure could cause numerous problems. With the increase in structure size, it will be harder for the virus to anchor to the gaps, and the virus will also have fewer effects on the scattering field of structures, i.e., leading to low sensitivity. The structure and size of nanodots in this article make a good balance between response ability and the size of the detection region.

## 4. Conclusion

In conclusion, we have demonstrated a detection method for virus-like particles with low concentration. A split-ring nanocavity structure is designed on the Si substrate as the detection unit. Simulation results in FDTD show a resonant peak under a wavelength of 558 nm under X polarization, which reveals the transverse mode with energy localized at two gaps. When a virus is attached with one gap and coated with a 10 nm Au layer, a unique asymmetric plasmonic mode will be generated at 670 nm wavelength, resulting in an asymmetric hot spot distribution. The far-field asymmetric features for units that contain viruses are successfully detected experimentally using the indirect polarization parameter sinδ.

An algorithm based on the extended Laplace operator has then been created to re-correct the background split-ring signal. After the convolution, an intensity decrease is revealed distinctly at the units containing viruses in the modified image. To increase the accuracy of the virus detection, we generated another asymmetry parameter from the asymmetric pixel values along the longitudinal orientation and obtained results from the extended Laplace image. The parameter gives an accurate judgment of virus existence on a large scale with a threshold absolute value of 7.

This structure shows an evident directivity of attachment points for viruses, which simplifies the analysis process for asymmetry. From theoretical calculation, our method provides high sensitivity to a virus-like particle with low concentration compared with the conventional detection method and our former work. In this article, the detection limit of our system reached 56 vp/150µL under a standard sample capacity. In addition, our method is compatible with other chemical process, such as surface modification, to achieve detection specificity. We believe this detection method shows a promising future for monitoring water impurities and the detection of bioparticles at a large scale.

## Data Availability

Data underlying the results presented in this paper are not publicly available at this time but may be obtained from the authors upon reasonable request.

## Supplemental document

See Supplement 1 for supporting content.
