# Supplementary material for: Asymmetric parameter enhancement in the split-ring cavity array for virus-like particle sensing
Source: Biomed Opt Express. 2023 Feb 22;14(3):1216–27. doi: 10.1364/BOE.483831 (PMC10026587; doi:10.1364/BOE.483831)
Supplement: Supplementary file 1 [file boe-14-3-1216-s001.pdf]

Supplemental Document

Biomedical Optics EXPRESS

Asymmetric parameter enhancement in the split-ring cavity array for virus-like particle sensing: supplement

XIAO JIN,[1,4] LU XUE,[1,4] SHENGWEI YE,[2] WEIQING CHENG,[2] JAMIE JIANGMIN HOU,[3] LIANPING HOU,[2] JOHN H. MARSH,[2] MING SUN,[1] XUEFENG LIU,[1] JICHUAN XIONG,[1,5,6] AND BIN NI[1,5,*]

[1] *School of Electronic and Optical Engineering, Nanjing University of Science and Technology, Nanjing 210094, China*
[2] *James Watt School of Engineering, University of Glasgow, Glasgow, G12 8QQ, UK*
[3] *Department of Medicine, University of Cambridge, Hills Road, Cambridge, CB2 0QQ, UK*
[4] *Co-first authors*
[5] *Co-last authors*
[6] *jichuan.xiong@njust.edu.cn*
[*] *nibin@njust.edu.cn*

This supplement published with Optica Publishing Group on 22 February 2023 by The Authors under the terms of the Creative Commons Attribution 4.0 License in the format provided by the authors and unedited. Further distribution of this work must maintain attribution to the author(s) and the published article's title, journal citation, and DOI.

Supplement DOI: https://doi.org/10.6084/m9.figshare.22085765

Parent Article DOI: https://doi.org/10.1364/BOE.483831

ASYMMETRIC PARAMETER ENHANCEMENT IN THE SPLIT-RING CAVITY ARRAY FOR VIRUS-LIKE PARTICLE SENSING: SUPPLEMENTAL DOCUMENT

XIAO JIN,[1, †] LU XUE,[1, †] SHENGWEI YE,[2] WEIQING CHENG,[2] JAMIE JIANGMIN HOU,[3] LIANPING HOU,[2] JOHN H. MARSH,[2] MING SUN,[1] XUEFENG LIU[1], JICHUAN XIONG[1, *] AND BIN NI[1, *]

[1] *School of Electronic and Optical Engineering, Nanjing University of Science and Technology, Nanjing 210094, P. R. China*
[2] *James Watt School of Engineering, University of Glasgow, Glasgow, G12 8QQ, UK*
[3] *Department of Medicine, University of Cambridge, Hills Road, Cambridge, CB2 0QQ, UK*
†Contributed equally
**jichuan.xiong@njust.edu.cn , nibin@njust.edu.cn*

This file includes:

I. Theory and calculation of PIMI
II. Diagram of PIMI system and PIMI initial images of split-ring
III. Simulation and experimental results for different sample statuses

I. Theory and calculation of PIMI

PIMI system [1] is a method to observe birefringence in samples and image indirect parameters at a large scale. Considering a birefringent position on the sample, the phase difference between fast axis and slow axis is

$$\delta = 2\pi L\Delta n/\lambda. \tag{S1}$$

Here L is light path and λ is wavelength, and the polarization ellipse orientation angle ϕ is defined as angle between fast axis and X axis. In PIMI system, a rotating polarizer with angle of θ_i is placed before the sample, with reflected light from the sample sequncingly go through a quarter wave plate and a 45° polarizer. The output Intensity are

$$I_i = \frac{1}{2}I_0\left[1 + \sin\delta \ \ \sin2(\theta_i - \phi)\right] \tag{S2}$$

Where I_i (the subscript i represents the number of polarization rotation angles) indicates the pixel intensity. I_0 is the average intensity under all polarization states. $sin\delta$ represents the sine of the phase difference between two orthogonal polarization components. θ_i is the polarization angle of the linearly polarized incident beam and ϕ is the polarization ellipse orientation angle of the reflected beam from the sample. By expanding Eq. (S2) trigonometrically, it can be reformulated in the following form:

$$I_i = a_0 + a_1 sin2\theta_i + a_2\cos2\theta_i. \tag{S3}$$

$$a_0 = \frac{1}{2}I_0, a_1 = \frac{1}{2}I_0\sin\delta\cos2\phi, a_2 = -\frac{1}{2}I_0 sin\delta sin2\phi. \tag{S4}$$

With a total number of steps N = 180°/18°, a_0, a_1 and a_2 can be calculated as:

$$a_0 = \sum_{i=1}^{N}\frac{1}{N}I_i, a_1 = \sum_{i=1}^{N}\frac{2}{N}I_i sin\alpha_i, a_2 = \sum_{i=1}^{N}\frac{2}{N}I_i cos\alpha_i \tag{S5}$$

Thus, the PIMI parameters, sinδ and ϕ can be extracted by utilizing the above equations.

$$sin\delta = \frac{\sqrt{a_1^2 + a_2^2}}{a_0}, \phi = \frac{1}{2}arccos\left(\frac{a_1}{\sqrt{a_1^2 + a_2^2}}\right) \tag{S6}$$

II. Diagram of PIMI system

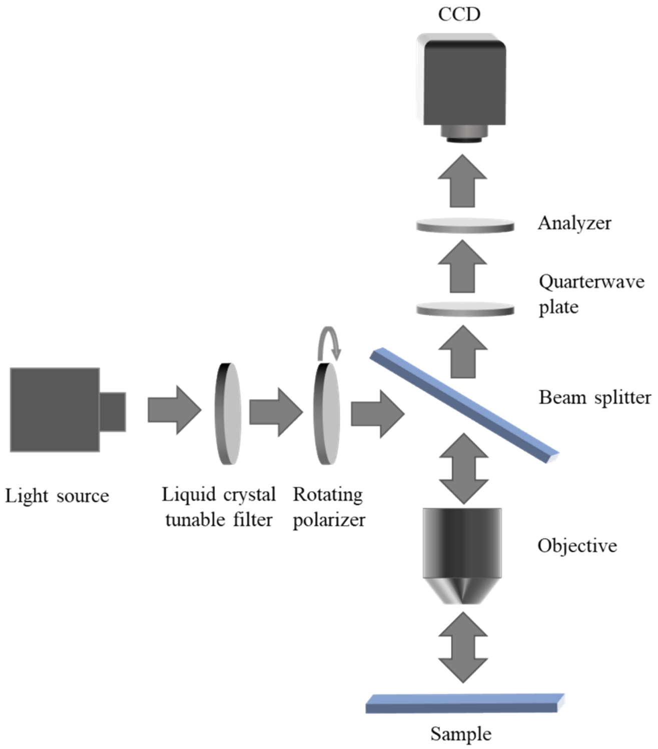

Fig. S1. Diagram of measurement using the PIMI system.

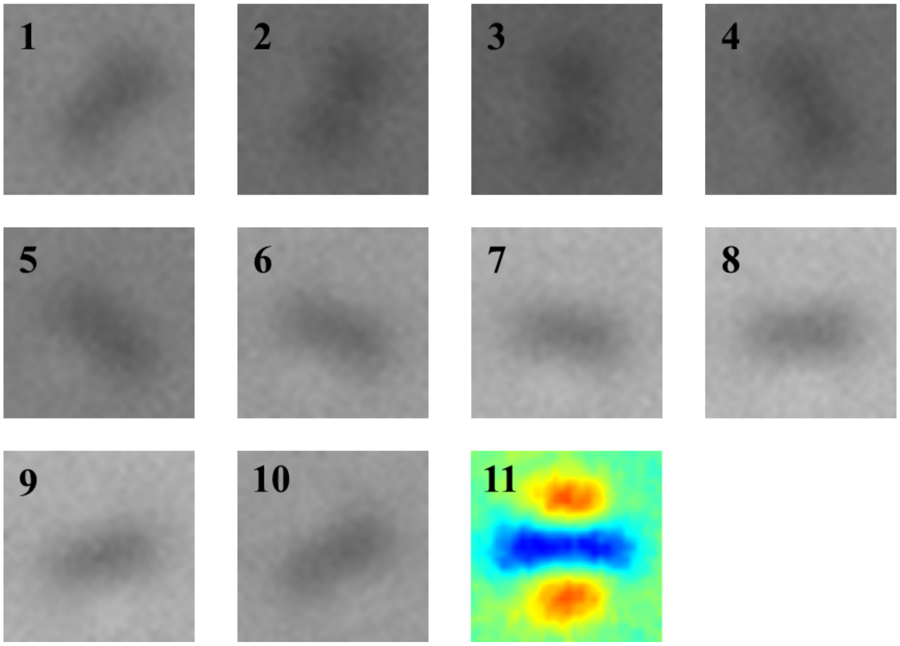

Fig. S2. 1-10: 10 PIMI initial images of split-ring under a rotating illumination with a step of 18°, 11: PIMI sinδ image

III. Simulation and experimental results for different sample statuses

In the manuscript, several sample processing steps are proposed to enhance the asymmetric signal. The sample could be classified into these statuses:

1. A bare split-ring.
2. A split-ring attached with a virus.
3. A bare split-ring covered with an Au layer.
4. A split-ring attached with a virus and covered with an Au layer.

The basic logic we tried to express in the manuscript could be simply conclude as follows in Fig. S3. A transverse mode is generated for a bare split-ring. When a virus is attached to the split-ring, as shown in Fig. S3 (b), barely any influences are generated to the transverse mode, which means a weak sensing ability. To enhance the asymmetry caused by the virus, a layer of Au is deposited. The basic transverse mode for the bare split-ring covered with the Au layer does not change. For the status 4, however, this mode cannot be held any more. Due to the physical connection of metal at the virus side, the mode symmetry is then strongly broken. In Fig. S3 (d), we could clearly recognize an asymmetry along the longitudinal direction, which represents the existence of the virus.

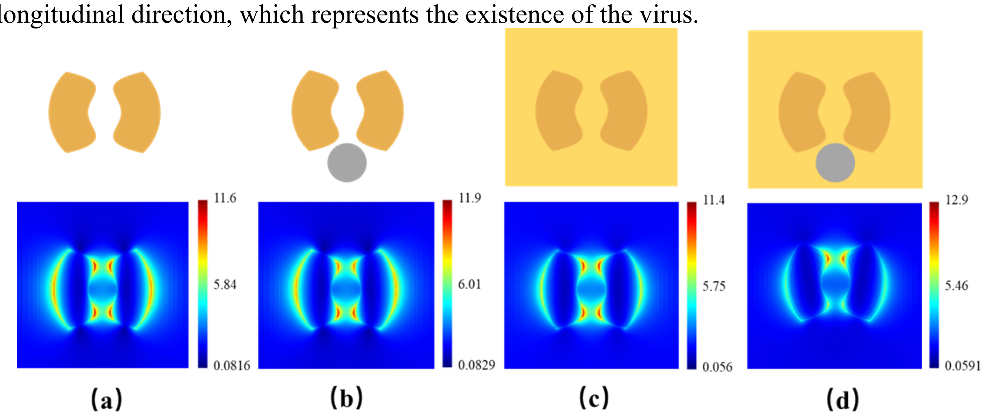

Fig. S3. Electric field distributions on the top surface of the structure for the status 1 to 4 under a X-polarized plane wave. (a) a bare split-ring. (b) a split-ring attached with a virus. (c) a bare split-ring covered with an Au layer. (d) a split-ring attached with a virus and covered with an Au layer.

The influences of this near-field physical mechanism would also be characterized at a certain height, where the electromagnetic waves propagate away from the metal surface and could be detected by the far-field techniques, i.e., the PIMI method mentioned in the manuscript. With a monitor set at the height of 100 nm from the top surface of the split-ring in the simulation, we could directly establish the connection between the near-field results and far-field polarization parameter images.

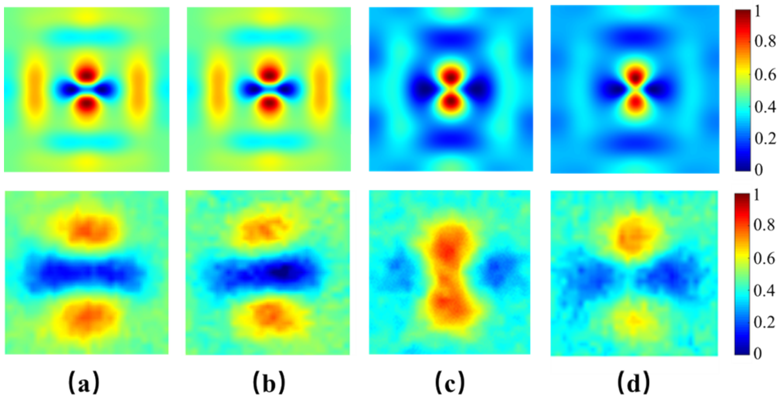

Fig. S4. Simulation and experimental PIMI results. (a) a bare split-ring. (b) a split-ring attached with a virus. (c) a bare split-ring covered with an Au layer. (d) a split-ring attached with a virus and covered with an Au layer. The upper parts are simulation results and the lower parts are experimental results.

The simulated sinδ results from Fig. S4(a) to (d) correspond to the near-field results from status 1 to status 4 in Fig. S3, respectively. For the bare split-ring, the transverse dipoles in the sinδ image connect with each other and form a strip, which indicates an asymmetry for near-field modes along X and Y direction. When a virus is attached, the sinδ pattern does not show too much mutation. The experimental results for these two statuses could match the simulation results with a transverse strip.

However, the sinδ pattern would alter to another form, where the longitudinal dipoles connect, rather than the transverse dipoles. This phenomenon is generated by interference between the scattering mode of the split-ring and the substrate reflection[2, 3]. Under this theory, the far-field electric field distribution we can detect could be transferred into other patterns when the substrate changes, i.e., a Si substrate and an Au substrate. This variation of the sinδ pattern is also observed in the experimental results in Fig. S4 (a) and (c). At last, for a sample site at status 4, an obvious asymmetry is generated along longitudinal direction both in the simulation and the experiment, i.e. Fig. S4 (c) and (d), which reflects the near-field mode asymmetry caused by the virus-attachment similar as in Fig. S3 (c) and (d).

For all four processing statuses of the sample, the experimental sinδ results agree well with the corresponding simulation results. In fact, the simulated PIMI results in Fig. S4 and simulated electric field distributions in Fig. S3 are acquired at different heights in the same simulations. That means these results share the same reliability. Thus, we believe that the uniformity between PIMI results for simulations and experiments in Fig. S4 can prove the change of near-field mode distribution caused by the virus.

References

1. X. Liu, B. Qiu, Q. Chen, Z. Ni, Y. Jiang, M. Long, and L. Gui, “Characterization of graphene layers using super resolution polarization parameter indirect microscopic imaging,” Optics express **22**(17), 20446-20456(2014).
2. P. T. Lyu, Q. Y. Li, P. Wu, C. Sun, B. Kang, H. Y. Chen, and J. J. Xu, “Decrypting material performance by wide-field femtosecond interferometric imaging of energy carrier evolution,” Journal of the American Chemical Society **144**(30), 13928-13937(2022).
3. S. Lin, Y. He, D. Feng, M. Piliarik, and X. W. Chen, “Optical Fingerprint of Flat Substrate Surface and Marker-Free Lateral Displacement Detection with Angstrom-Level Precision,” Physical Review Letters **129**(21), 213201(2022).
